# Development of a Prediction Model for Survival Time in Esophageal Cancer Patients Treated with Resection.

**DOI:** 10.23889/ijpds.v7i3.2097

**Published:** 2022-08-25

**Authors:** Lyndsay Harrison, Alyson Mahar, Natalie Coburn, Randy Boyes, Michael Pugliese, Carolyn Compton, Gail Darling, Laura Davis, Kathleen Decker, Vaibhav Gupta, Biniam Kidane, Douglas Manuel, Jolie Ringash, Donna Turner, Amy Hsu

**Affiliations:** 1 University of Manitoba; 2 ICES; 3 University of Toronto; 4 Queen's University; 5 Arizona State University; 6 Dalhousie University; 7 McGill University; 8 University of Ottawa

## Objectives

Accurate estimates of survival guide decision-making for patients and oncologists. Advances in the capacity to measure complex tumour biology and patient factors allow for concurrent consideration of clinical, pathological, molecular, and biological markers for prognostication. Clinical prediction tools are a mechanism to combine and personalize these increasingly large amounts of complex information for prognostication.

## Approach

We describe the process of linking routinely collected health data, cancer registry, and pathology report data in two provinces to develop (Ontario, Canada) and validate (Manitoba, Canada) a clinical prediction tool in esophageal cancer. We compared the performance of a base model restricted to patient and disease characteristics available prior to surgical resection (e.g., age, sex, histology, comorbidities), and a more complex model including pathology specimen details (e.g., tumour stage). Cox proportional hazards models were fit to predict death at three years following resection. Internal and external validity was assessed using overall calibration and optimism corrected c-statistics. Equity was assessed through calibration in predefined patient subgroups.

## Results

2124 patients who underwent surgical resection for esophageal cancer between May 1, 2004 and June 30, 2016 for whom a pathology record was available were included in the study cohort. Median age was 66, with 80% males and 85% adenocarcinomas. Survival data were available until March 31, 2020. The model with pathology data had superior discrimination and calibration (calibration slope of 1.02 and intercept -0.01, and optimism-corrected c-statistic 0.77), compared to the base model (calibration slope of 0.95, intercept 0.02, and c-statistic 0.60). External validation is ongoing.

## Conclusion

Our study demonstrates that prediction models for cancer prognosis built solely on data from health administrative databases may be unreliable. The addition of high-quality pathology report data from electronic medical records or population-based cancer registries is necessary for accurate estimation. Our work provides a framework for combining administrative and clinical data which could be applied to the development of other clinical prediction models.

